# JAK1/2 Inhibitor Baricitinib Improves Skin Fibrosis and Digital Ulcers in Systemic Sclerosis

**DOI:** 10.3389/fmed.2022.859330

**Published:** 2022-06-06

**Authors:** Zhanying Hou, Xuehan Su, Guangming Han, Ruzeng Xue, Yangxia Chen, Ye Chen, Huan Wang, Bin Yang, Yunsheng Liang, Suyun Ji

**Affiliations:** ^1^Department of Dermatology, Dermatology Hospital, Southern Medical University, Guangzhou, China; ^2^Department of Dermatology, Shenzhen Longhua District Central Hospital, Shenzhen, China; ^3^The First School of Clinical Medicine, Southern Medical University, Guangzhou, China; ^4^Department of Rheumatology, Dermatology Hospital, Southern Medical University, Guangzhou, China

**Keywords:** baricitinib, JAK inhibitor, systemic sclerosis, skin fibrosis, digital ulcers

## Abstract

**Background:**

Systemic sclerosis (SSc) is a rare disabling connective tissue disease with few available treatment options. Diffuse cutaneous systemic sclerosis (dcSSc) is associated with high mortality. A previous experiment has shown that JAK2 inhibitor can significantly improve skin fibrosis in bleomycin (BLM)-induced murine model, including reducing dermal thickening and collagen accumulation. We aimed to describe the efficacy of oral JAK1/2 inhibitor baricitinib in SSc patients, especially focusing on skin fibrosis and microvascular manifestations.

**Methods:**

We described the different effects of oral selective JAK1, JAK2, or JAK3 inhibitor treatment in a BLM-induced skin fibrosis mouse model. Furthermore, 10 adult patients with dcSSc were treated with baricitinib. We assessed the changes in modified rodman skin score (mRSS) and digital ulcer net burden at week 12 and 24 from baseline. We also compared the absolute changes in scores on the Scleroderma Health Assessment Questionnaire (SHAQ) and a total score on the St. George's Respiratory Questionnaire (SGRQ) over a 24-week period.

**Results:**

In the experimental mouse model of skin fibrosis, a JAK1 and JAK2 inhibitor ameliorated skin fibrosis, and a JAK2 inhibitor had the most obvious effect. Treatment with the JAK2 inhibitor also blunted the capillary rarefaction. We demonstrated that skin fibrosis and digital ulcers were significantly relieved in 10 SSc patients treated with baricitinib. The mRSS significantly improved at week 12 from baseline, with a mean change in mRSS of −8.3 [95% confidence interval (CI), −12.03 to −4.574; *p* = 0.0007] and improved greater at week 24 to −11.67 (95% CI, −16.84 to −6.496; *p* = 0.0008). Among the four patients with digital ulcers (DU), three were completely healed at week 24, the number of ulcers in another patient was significantly reduced, and there was no patient with new ulcers. Only one adverse event (AE) of herpes zoster was observed.

**Conclusions:**

Our results indicate that selective JAK1 and JAK2 inhibitor alleviates skin fibrosis, and oral JAK1/2 inhibitor baricitinib is a potentially effective treatment for dcSSc patients with skin fibrosis and DU. Baricitinib was well-tolerated by most patients in this study. Additional large clinical trials are needed to confirm our pilot findings.

**Chinese Clinical Trial Registry Number:**

ChiCTR2000030995.

## Introduction

Systemic sclerosis (SSc) is a rare connective tissue disease affecting the skin and internal organs, which is characterized by vasculopathy, inflammation, and fibrosis (1). Overall prognosis remains poor, and diffuse cutaneous systemic sclerosis (dcSSc) is associated with high mortality due to early visceral involvement, representing a rapidly progressing disorder affecting a large skin area and compromising one or more internal organs (2). As the characteristics of dcSSc, rapid progressive pain, digital ulcers (DU), Raynaud's phenomenon, and skin tension are the symptoms that seriously affect the quality of patients' life. Substantial skin involvement or early rapid progressive skin fibrosis is associated with increased mortality and internal organ involvement (3). To date, treatment of dcSSc is very challenging. The efficacy and safety of methotrexate (MTX), cyclophosphamide (CYC), and mycophenolate mofetil (MMF) in dcSSc are still controversial (4). Currently, nintedanib has been approved for the treatment of SSc-associated interstitial lung disease (ILD) (5), and studies have shown that tocilizumab, lenabasum, abatacept, and riociguat could improve the modified Rodnan skin score (mRSS) but did not show a statistically significant improvement compared to placebo (6–9). Recently, a 24-week phase II study has shown that romilkimab had significant effects on skin changes in early dcSSc, but it still requires further confirmation (10). Therefore, there is a growing unmet need for novel, effective therapeutics in dcSSc.

The Janus kinase (JAK) and signal transducers and activators of transcription (STAT) signaling pathways play an important role in pro-inflammatory or pro-fibrotic signals to target cells during systemic sclerosis (SSc), including inflammatory cells, endothelial cells, and fibroblasts (1, 11, 12). Tissue damage associated with SSc is caused by inflammatory cytokines, such as interleukin (IL)-4, IL-13, IL-6, IL-12, and IL-23, and involves the dysregulation of Th2-dominant immunity, inflammation, and fibrotic responses, effects of which are partly mediated by JAKs (1, 12, 13). Furthermore, transforming growth factor-β (TGF-β), a core pathway of fibroblast activation in fibrotic diseases, including SSc (14), is a potent pro-fibrotic cytokine promoting migration and differentiation into myofibroblasts of fibroblast, upregulating the synthesis of ECM components, and inducing adhesion and fibrosis through various pathways or interactions with other mediators (15). It has been demonstrated to activate p-JAK2 in fibrosis (16). Thus, JAKs can be the targets for anti-fibrotic and anti-inflammatory therapies in SSc. Nevertheless, the effects of JAK inhibition on vascular manifestations have not been investigated so far.

Several JAK inhibitors (JAKi), including tofacitinib, baricitinib, and ruxolitinib, are already being investigated for the treatment of various autoimmune diseases (17). JAK inhibitors have been approved to treat rheumatoid arthritis (RA) (18–20) for many years, whereas the efficacy of JAK inhibitors in SSc remains unclear. Tofacitinib, an oral pan-JAK inhibitor, has improved cutaneous stiffness and the healing of DU in a 27-year-old SSc male patient (21). Furthermore, tofacitinib and baricitinib have been shown to improve ILD in amyopathic dermatomyositis (ADM) (22, 23) and RA (19). Therefore, JAK inhibitors may be promising therapeutic agents for SSc and SSc-ILD.

Previous studies have shown that targeting JAK2 can improve skin fibrosis in mouse models of SSc (24). Therefore, we used a bleomycin (BLM)-induced dermal fibrosis mouse model to directly study the effects of JAK inhibition in scleroderma and observe and compare its efficacy to provide a theoretical basis for the selection of medications in the clinical treatment of SSc. Then, we conducted a clinical trial to study the efficacy of oral JAK1/2 inhibitor baricitinib in patients with dcSSc, especially focusing on skin fibrosis and microvascular manifestations. Our clinical research is possibly one of the first to specifically focus on the efficacy of the JAK1/2 inhibitor (baricitinib) to treat skin fibrosis and DU in patients with dcSSc.

## Methods

### Mice and JAK Inhibitors

Eight-week-old *C57Bl/6* female mice were purchased from the Guangdong Medical Laboratory Animal Center (Guangzhou, China). The JAK1 inhibitor, itacitinib (catalog HY-16997, MedChemExpress); JAK2 inhibitor, TG101209 (catalog HY-10410, MedChemExpress); and JAK3 inhibitor, JANEX-1 (catalog HY-15508, MedChemExpress) were dissolved in DMSO (catalog D12345, Thermo Fisher Scientific) along with Tween80, PEG300 (catalog P4780, Sigma-Aldrich), and saline according to the manual.

### BLM-Induced Skin Fibrosis Model

For BLM-induced skin fibrosis, 8-week-old female mice received repeated subcutaneous injections of 2 usp/ml BLM (100 μl) in selected 1 cm^2^ areas on the upper back every other day for 28 days. Control mice were injected with equal volumes of PBS. In three subsets of the BLM-treated group, mice were treated with oral gavage of JAK1, JAK2, or JAK3 inhibitor (30 mg/kg/day), respectively, for 21 days starting from the 8th day. Meanwhile, other mice were treated with a vehicle with equal volumes of the inhibitor.

### Histologic Analysis

The BLM- or PBS-injected skin areas were fixed in formalin, embedded in paraffin, and stained with hematoxylin and eosin or Masson's trichrome (Sigma-Aldrich, Germany). The dermal thickness was visualized using a Nikon Eclipse 80i microscope (Nikon) and analyzed at three different sites in each mouse in a blinded manner.

### RNA Isolation and Quantitative Real-Time PCR

Total RNA from the fibrotic skin of the mice was extracted using the Trizol reagent and reverse transcribed using Takara PrimeScript™ RT Master Mix. Gene expression was quantified by SYBR green real-time PCR on the CFXConnect quantitative PCR System in triplicates.

### Immunofluorescence

After deparaffinization, sections were incubated using a citrate buffer (catalog C9999, Sigma-Aldrich) for 15 min in a microwave. After the non-specific binding sites were blocked with 3% bovine serum albumin (catalog B2064, Sigma-Aldrich) in PBS for 1 h at room temperature, the sectionswere incubated with an anti-CD31 antibody (catalog BS1574, 1:100, Bioworld) overnight at 4°C, washed in PBS, and incubated with Alexa Fluor 647-labeled secondary antibody (catalog ab150063, 1:200, Abcam) for 1 h at room temperature. Finally, slides were mounted with DAPI-Aqueous, Fluoroshield (catalog ab104139, Abcam). Immunofluorescence images were captured and analyzed on a Nikon A+ confocal microscope.

### Trial Design and Participants

This clinical trial was a single-center, open-label clinical trial registered in the Chinese Clinical Trial Registry (Reg No. in ChiCTR: ChiCTR2000030995). Patients were recruited at the Dermatology Hospital of Southern Medical University from January to October in 2020. The enrolled patients were treated with baricitinib (2 or 4 mg daily).

### Clinical Trial Participants

All patients were at least 18 years old at enrollment and fulfilled the American College of Rheumatology/European League Against Rheumatism (ACR/EULAR) classification criteria (25) and the classification according to LeRoy and Medsger (26, 27) for ILD. Patients were excluded if they had other connective tissue diseases, underlying cancer, a history of heart failure, a concomitant infection, or a liver aminotransferase level greater than twice the upper limit of the normal range. Pregnant, nursing, and unwilling or unable to use contraception women were excluded. The enrolled patients were treated with baricitinib (2 or 4 mg daily).

### Clinical Trial Procedures

All enrolled patients were followed up for 24 weeks. The same physician, experienced in skin scoring, scored a single patient throughout the duration of the study at baseline, week 12, and week 24 to prevent interobserver variability. Demographics and clinical features were recorded at baseline. The efficacy and safety of baricitinib were evaluated at week 12 and 24. Modified rodman skin score (mRSS) and digital ulcer net burden were estimated at each visit. Patients with a decrease in mRSS of >5 points and ≥25% from baseline were classified as improvers (28). Given the high mortality associated with ILD and the inability to reverse the fibrotic process once established, high-resolution computed tomography (HRCT) of the lungs was performed for 10 patients at baseline and repeated for 9 patients at week 24. Changes from baseline to week 24 based on the Scleroderma Health Assessment Questionnaire (SHAQ) - Visual Analog Scale (VAS) and St. George's Respiratory Questionnaire (SGRQ) were calculated. Adverse events were also recorded. Safety was assessed by the monthly monitoring of review of systems, complete blood cell count with a differential, complete metabolic panel, and fasting lipid panel.

### Clinical Trial Outcomes

The primary endpoint was the change in mRSS from baseline to week 24. Skin fibrosis was also analyzed *via* the percentage of patients (%) with an improvement in mRSS of <25, 25–50, 51–75, and >75% from baseline to week 24. The secondary endpoints were absolute changes in numbers of DU, scores on SHAQ, and total score on the SGRQ from baseline to week 24. DU was defined as present (active) in the case of epithelialization loss on the distal surface of the finger caused by skin rupture or ischemia that is not located on the surface of subcutaneous calcification or joint extensor. Data on both the number and position of cutaneous DU on any finger were collected at the baseline and each study visit (29). New lesions were particularly noted. SHAQ-VAS included overall disease severity, pain severity, gastrointestinal function, breathing function, vascular function (Raynaud's phenomenon), and digital ulcer impact on activity. The SGRQ was used to measure health-related quality of life in patients with ILD. It was a 50-item self-assessment questionnaire used to assess the health-related quality of life in patients with respiratory disease. It included three main domains (symptoms, activity, and impact) and was scored from 0 to 100, with higher scores indicating poorer health-related quality of life (30). To date, no minimal clinically important difference in SGRQ total score in patients with SSc-associated ILD has been established; however, a change of 4 points or more may represent a meaningful change in patients with idiopathic pulmonary fibrosis alone (31).

### Statistical Analysis

In this study, statistical analyses were performed using GraphPad Prism 7.0 (La Jolla, CA 92037, USA). Data were presented with mean ± standard deviation (SD), and comparisons among groups were analyzed by using Student's *t*-test or one-way analysis of variance (ANOVA). For all comparisons, *p*-values of < 0.05 were considered statistically significant.

### Study Approval

The study protocols were performed in accordance with the Declaration of Helsinki and were reviewed and approved by the Institutional Ethical Committee of Dermatology Hospital of Southern Medical University. All patients provided written informed consent prior to enrollment in the trial. Permission was obtained from patients for the publication of photos.

## Results

### Inhibition of JAK1 and JAK2 Alleviates Skin Fibrosis, With JAK2 Inhibitor Promoting Microvascular Reconstruction in BLM-Induced Skin Fibrosis Model

**Figure 1:**
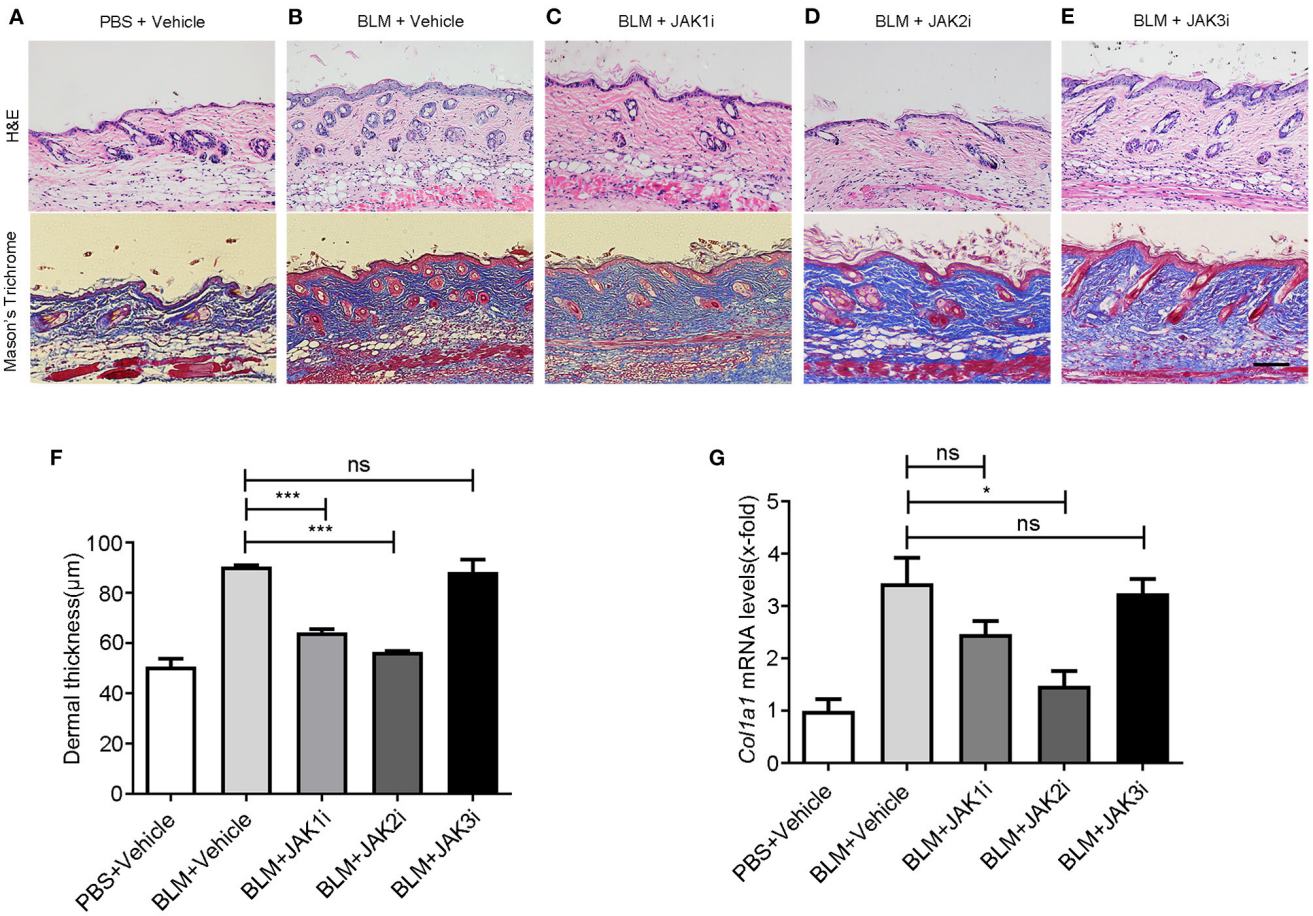
Changes in pathology and gene expression after the treatment with JAK inhibitors in BLM-induced skin fibrosis model **(A–E)** Representative images of skin sections stained with H&E (upper pictures) and Mason's trichome (lower pictures) of PBS-treated mice exposed to vehicle, BLM-treated mice exposed to vehicle, and BLM-treated mice receiving itacitinib (JAK1i), TG101209 (JAK2i), and JANEX-1 (JAK3i), respectively. Scale bar: 200 μm. **(F)** Dermal thickness was measured to determine the degree of dermal fibrosis, demonstrating that JAK2 inhibitor treatment reduces dermal fibrosis the most. **(G)** Relative mRNA levels of *Col1*α*1*. Values are presented as the mean of fold change transcripts ± SEM. *0.01 ≤ *p* < 0.05; *** *p* < 0.001 (unpaired Student's *t*-test). Three replicate experiments were conducted for a sum of six mice per group.

To determine which JAK is the best target and to assess the role of selective JAKis on fibrosis and vasculopathy, we used a BLM-induced murine model of fibrosis with features of human scleroderma, including similar dermal sclerosis, collagen synthesis, and inflammatory infiltration (32). Bleomycin injection led to skin fibrosis. Then, we compared the effects of JAK1, JAK2, and JAK3 inhibition in BLM-induced skin fibrosis using selective JAK1, JAK2, and JAK3 inhibitors, respectively. JAK inhibition was well-tolerated in all experiments, as indicated by constant body weight, the normal texture of the fur, and normal activity. JAK1 and JAK2 but not JAK3 inhibitors alleviated skin fibrosis in different aspects, such as morphology and dermal thickness, and the JAK2 inhibitor alleviated skin fibrosis the most (Figures 1A–E), demonstrating that JAK inhibition prevented fibrosis in a BLM-induced mouse model of scleroderma. In the group of mice receiving the JAK2 inhibitor, we found a 37.5% decrease in skin thickness (Figure 1F). Furthermore, the upregulation of *Col1a1* mRNA levels was reduced after selective JAK2 inhibitor treatment (Figure 1G), indicating that JAK2 inhibition decreased collagen production. However, we did not detect significant inhibitory effects of either selective JAK1 or JAK3 inhibitor treatment on *Col1a1* gene transcription.

**Figure 2:**
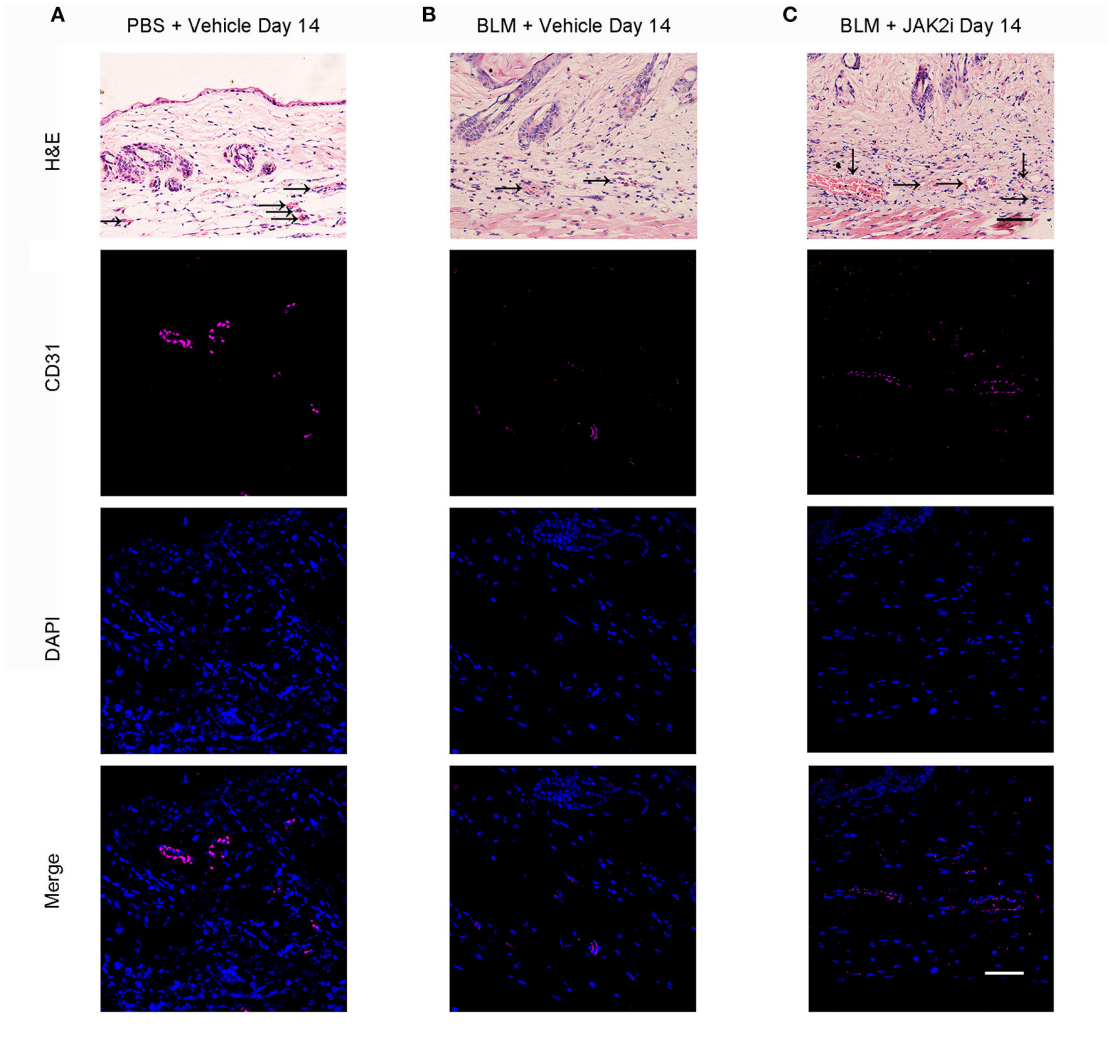
Changes of capillary loss after the treatment with JAK inhibitor in BLM-induced skin fibrosis model on day 14 Representative H&E (scale bar: 50 μm) and immunofluorescence (scale bar: 50 μm) images stained for CD31 (endothelial cells, purple) and DAPI (nuclear staining, blue) of skin sections from PBS-treated mice exposed to vehicle **(A)**, BLM-treated mice exposed to vehicle **(B)**, and BLM-treated mice receiving 30 mg/kg/day JAK2 inhibitor **(C)** on day 14. Scale bar: 50 μm. Three replicate experiments were conducted for a sum of six mice per group.

The loss of capillaries is one of the features of scleroderma and the Fra2 transgenic mice SSc mouse model (33). BLM induces skin fibrosis and vascular damage. With the JAK2 inhibitor treatment, the capillary loss was reduced, and the numbers of vessels in the dermis of JAK2i-treated mice increased at days 14 (Figures 2A–C) and 28 (Supplementary Figures 1A–C).

### Clinical Trial Participants

**Figure 3:**
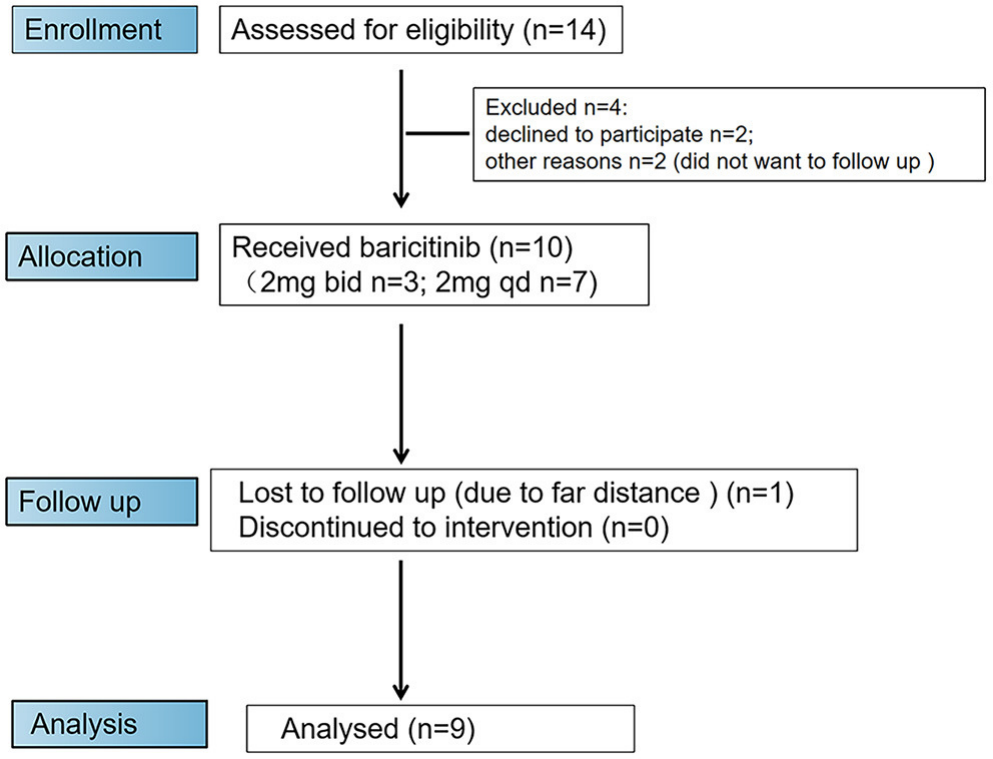
Flow diagram of enrollment procedure. Ten patients were enrolled in the trial and received baricitinib. One patient dropped out of the trial due to the inability to attend trial visits at week 24.

Of 14 initial candidates, 10 patients were eligible to participate in the trial (Figure 3). The demographic data and clinical characteristics of 10 patients (six females and four males) enrolled in the trial are listed in Table 1. Most participants were female [6 (60.0%)], with a median age of 43.5 years (range, 25.0–58.0 years). The median disease duration was 19.5 months (range, 7.0–120.0 months), including 5 cases with <20 months of disease duration from the time of first non-Raynaud's phenomenon manifestation and five cases with ≥ 20 months of disease duration. Of 10 baricitinib-treated patients, three were previously refractory to conventional immunosuppressants (CsA or CYC), while six patients had an inadequate response to glucocorticoids (Table 1).

**Table 1:** Baseline information of SSc patients at enrollment.

**Patient**	**Gender**	**Age** **(years)**	**SSc** **course (months)**	**Systemic** **involvement**	**Previous treatment**	**Treatment** **after enrollment**	**ESR** **(mm/h)**	**Anti-Scl70** **(1 = positive; 0 = negative)**	**Baseline mRSS**
1	F	58	15	Digital ulcers + ILD + arthritis	None	Baricitinib 2 mg + nifedipine + NSIAIDs	60	1	18
2	F	50	15	ILD	Pred 15 mg (6 months) + Chinese herb	Baricitinib 4 mg for the first 12 weeks and then 2 mg	27	1	16
3	F	48	120	Digital ulcers + ILD + arthritis	CsA 100 mg (6 months), then Pred 15 mg (3 months) + pirfenidone + sildenafil	Baricitinib 2 mg + Pred 10 mg	12	1	36
4	F	39	36	Digital ulcers + ILD	CYC 150 mg (4 months), then Pred 25 mg (3 months) + PGE1 + nifedipine	Baricitinib 2 mg + Pred 10 mg	14	1	12
5	M	41	24	Digital ulcers	Pred 30 mg (6 months) + nifedipine	Baricitinib 4 mg + Pred 10 mg + nifedipine	20	0	24
6	M	61	60	ILD	CsA 100 mg (6 months), then Pred 15 mg (3 months) + tripterygium + nifedipine	Baricitinib 2 mg	25	1	50
7	M	32	50	ILD	Pred 15 mg (4 months) + tripterygium + tranilast	Baricitinib 2 mg	15	1	30
8	M	30	7	ILD	Pred 10 mg (6 months) + PGE1	Baricitinib 2 mg	3	1	36
9	F	25	12	ILD	Chinese herb	Baricitinib 2 mg	50	1	40
10	F	27	15	ILD	Pred 15 mg (3 months) + Chinese herb + PGE1	Baricitinib 4 mg + nifedipine	43	1	24

*CsA, cyclosporine; CYC, cyclophosphamide; ESR, erythrocyte sedimentation rate; F, female; M, male; Pred, prednisone; PGE1, prostaglandin E1; NSAIDs, non-steroidal anti-inflammatory drugs; Anti-Scl70, anti-topoisomerase I antibodies; ILD, interstitial lung disease; mRSS, modified Rodnan skin score*.

Of the patients, 90.0% (9/10) were positive for anti-topoisomerase antibodies. The skin involvement with a mean baseline mRSS was 28.6 (11.96 SD), and all four (40.0%) out of 10 patients had DU at baseline. According to the HRCT results of 10 patients at baseline, nine patients were diagnosed with SSc-ILD.

In this study, all patients were treated with baricitinib. Among them, three patients were treated with baricitinib 4 mg once daily, and seven patients were treated with baricitinib 2 mg once daily. Three (30.0%) out of 10 patients were treated with low-dose prednisone at baseline. After the initiation of the treatment with baricitinib, some patients continued to be treated with the original dose of prednisone, while others were treated with a reduced dose of prednisone or discontinued this treatment (Table 1). One case was withdrawn from the trial at week 24 due to the far distance and difficulties of reaching the center, and no patient was discontinued due to poor efficacy or side effects (Figure 3).

### Skin Fibrosis

**Figure 4:**
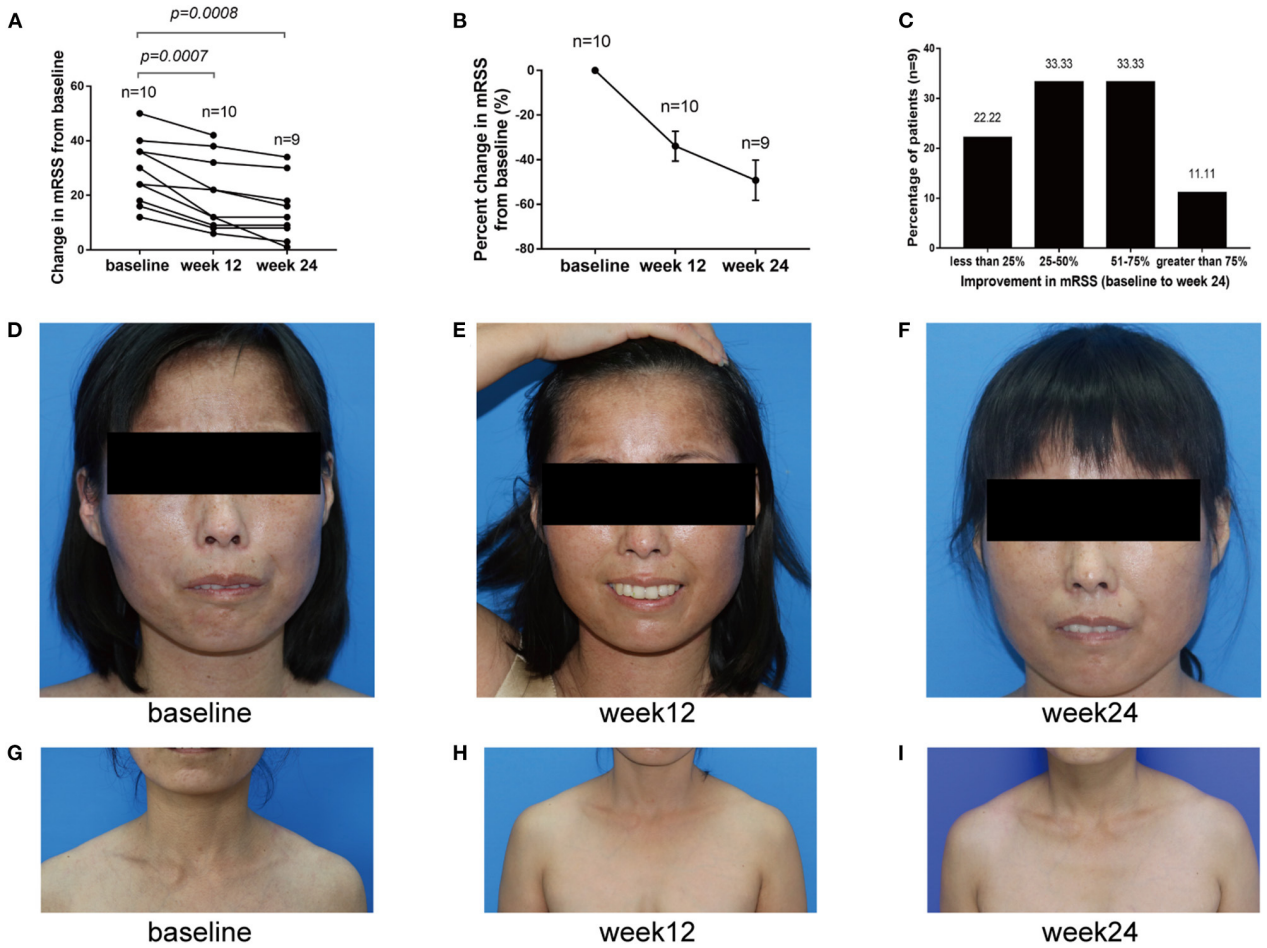
Change of mRSS from baseline to week 24. **(A)** mRSS changes from baseline to week 24. **(B)** Percent change from baseline to week 24 in mRSS. **(C)** mRSS responder rates. They indicated that <25, 25–50, 51–75 and >75% improvements in mRSS from baseline to week 24 were 22.22, 33.33, 33.33, and 11.11%, respectively. **(D–I)** Representative images of patients treated with baricitinib from baseline to week 24, from which we can infer that baricitinib significantly improved the patient's skin clinical symptoms.

Skin thickening in 10 baricitinib-treated patients was significantly decreased. The mRSS significantly improved at week 12 from baseline, with a mean (SD) change in mRSS of −8.3 (5.208) [95% confidence interval (CI), −12.03 to −4.574; *p* = 0.0007; *n* = 10] and improved greater at week 24 to −11.67 (6.727) (95% CI, −16.84 to −6.496; *p* = 0.0008; *n* = 9; Figure 4A). The mRSS of all 9 baricitinib-treated patients decreased by >5 points, and that of 7 (77.78%) cases decreased by ≥25% at week 24. The mean (±SE) percent decline in the mRSS score from baseline to week 24 occurred with reductions of 33.93% (±6.726) at week 12 and 49.23% (±8.985) at week 24 (Figure 4B). The frequency distribution of individual patient responses also showed an improvement in most participants. A responder rate analysis indicated that mRSS improved by ≥25% in 77.78% of the patients. Among them, mRSS improved by 25–50, 51–75, and >75% in 33.33, 33.33, and 11.11% of the patients, respectively (Figure 4C). And the skin clinical symptoms of these SSc patients were all significantly improved (Figures 4D–I).

### Digital Ulcers, Raynaud's Phenomenon, and SGRQ and SHAQ Scores

**Figure 5:**
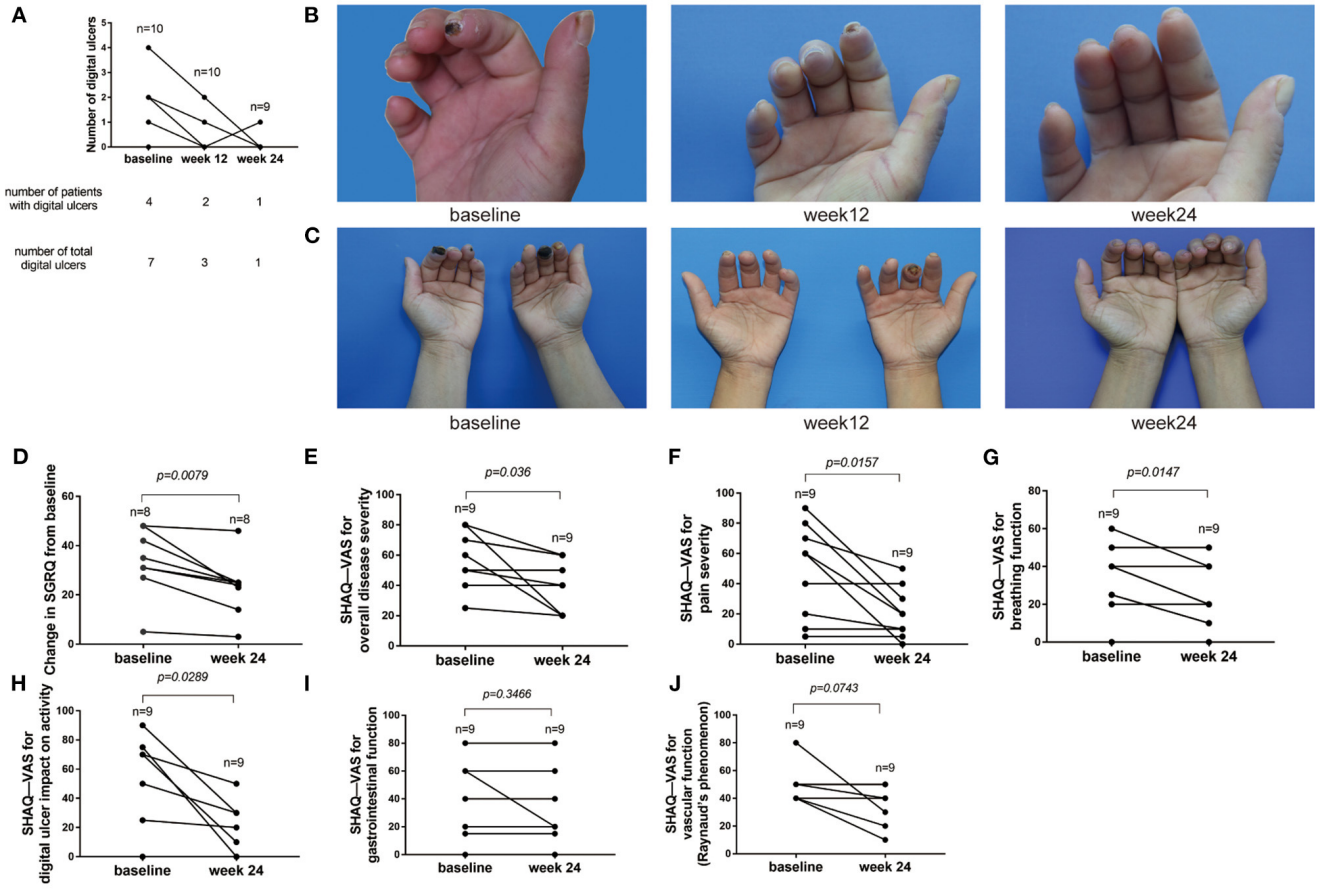
Changes of digital ulcers and SGRQ and SHAQ-Vas score from baseline to week 24. **(A)** Numbers of DU in 10 participants. Four (40.0%) patients had DU. Three patients were completely healed at week 24. One digital ulcer re-occurred in the same region at week 24 due to heavy manual work. **(B,C)** Representative images of DU treated with baricitinib from baseline to week 24. **(D)** SGRQ score from baseline to week 24. St. George's Respiratory Questionnaire (SGRQ). **(E)** SHAQ-VAS scores for overall disease severity from baseline to week 24. **(F)** SHAQ-VAS scores for pain severity from baseline to week 24. **(G)** SHAQ-VAS scores for breathing function from baseline to week 24. **(H)** SHAQ-VAS scores for DU impact on activity from baseline to week 24. **(I)** SHAQ-VAS scores for gastrointestinal function from baseline to week 24. **(J)** SHAQ-VAS scores for vascular function (Raynaud's phenomenon) from baseline to week 24. Scleroderma Health Assessment Questionnaire (SHAQ).

At baseline, 4 (40.0%) out of 10 patients had DU. No new DU were reported at week 12 and 24. Among four patients with DU at baseline, three patients completely healed at week 24. One patient with two DU healed at week 12; however, one digital ulcer re-occurred in the same region at week 24 due to the patient engaging in heavy manual work (Figure 5A). One ulcer in 1 patient healed at week 12. Four digital ulcers in 2 patients improved significantly at week 12 and completely healed at week 24 (Figures 5B,C). No concomitant medication for DU was used by those four patients during the 24 weeks. After the treatment with baricitinib, the respiratory symptoms of the eight patients with ILD were significantly improved, and the respiratory score decreased significantly. The mean (SD) change in SGRQ scores was −10.25 (7.888) (95% CI, −16.84 to −3.656; *p* = 0.0079; Figure 5D). However, by comparing the lung CT of the eight patients with ILD at baseline and week 24, only two were significantly improved, one was slightly aggravated, and the other five did not change (Supplementary Figures 2A–I). Among nine patients who completed the SHAQ-VAS questionnaire at baseline and week 24, most had significantly reduced SHAQ values at week 24, with the mean (SD) change in overall disease severity of −17.22 (20.17) (95% CI, −32.73 to −1.716; *p* = 0.0336), pain severity of −27.78 (27.28) (95% CI, −48.75 to −6.805; *p* = 0.0157), breathing function of −10 (9.682) (95% CI, 17.44 to −2.557; *p* = 0.0147), and digital ulcer impact on the activity of −26.67 (30.1) (95%CI, −49.81 to −3.527; *p* = 0.0289; Figures 5D–H). There was no significant difference in gastrointestinal [the mean (SD) of −4.444 (13.33); 95% CI, −14.69–5.804; *p* = 0.3466] and vascular function [Raynaud's phenomenon; the mean (SD) of −12.22 (17.87); 95% CI, −25.96–1.516; *p* = 0.0743; Figures 5I,J]. Although there was no significant difference in vascular function (Raynaud's phenomenon), the score decreased by more than 50% at week 24 in three cases (33.33%) out of nine patients (Figure 5J).

## Discussion

Our study is the first to highlight the therapeutic efficacy of JAK1/2 inhibitor baricitinib in treating skin fibrosis and microvascular manifestations of patients with dcSSC. We showed that selective JAK1 and JAK2 inhibitor ameliorated skin fibrosis, and JAK2 inhibitor alleviated skin fibrosis the most. Moreover, JAK2 inhibitor use resulted in a capillary loss in BLM-induced injury. In parallel, our clinical research is one of the first clinical trials to describe the efficacy of baricitinib, an oral JAK1/2 selective inhibitor, with a focus on skin fibrosis and microvascular manifestations, including DU and Raynaud's phenomenon, in dcSSc.

### Why JAK2 Inhibitor Could Alleviate Skin Fibrosis the Most in Mice Model

One reason could be that TGF-β1 signaling induces the phosphorylation and activation of JAK2, which then interacts with and phosphorylates STAT3 to induce fibrotic response (24). Nevertheless, recent findings have suggested that JAK1 might also be capable of activating STAT3 either directly (34) or indirectly *via* the transphosphorylation of JAK2 (35). An additional reason may be that JAK2 is the only member of the JAK family that pairs with itself. In this way, JAK2 controls the signaling of various cytokines and growth factors (36).

### Why JAK2 Inhibitor Could Be Useful in Microvascular Manifestations in Mice Model

The molecular mechanism underlying this phenomenon is still unknown. The primary role of JAK inhibitors is to block the function of multiple cytokines, such as type I and II cytokines (37). JAK2 can additionally control the signaling of various cytokines and growth factors, such as granulocyte-macrophage colony-stimulating factor (GM-CSF), IL-3, and IL-5 (36, 38). Based on the above-mentioned, we hypothesize that the effects of JAKis in skin fibrosis and microvascular dysfunction are associated with multiple inflammatory cytokines that contribute to SSc.

### The First Clinical Highlight—Therapeutic Efficacy of Baricitinib in Skin Fibrosis

In our study, most patients had an inadequate response to conventional immunosuppressants or glucocorticoids. Our results revealed that baricitinib can significantly improve skin fibrosis in dcSSc patients with progressive skin thickening and was better or at least as effective as glucocorticoids or intensive conventional immunosuppressants (CYC or CsA). Additionally, our observations of decreased skin fibrosis with JAK1/2 selective inhibitor baricitinib concur with the findings of prof. Zeng, who reported that pan-JAK inhibitor tofacitinib has been effective in refractory dcSSc patients with progressive skin thickness (28). Similarly, treatment with JAK inhibitors has provided positive effects in other sclerosing diseases, such as generalized morphea, eosinophilic fasciitis, and sclerodermatous graft-versus-host disease (39–41). Our data suggest that baricitinib is efficacious against skin fibrosis in dcSSc patients and highlight the need for additional study on JAK inhibitors for the treatment of fibrosing disorders.

### The Second Clinical Highlight—The Therapeutic Efficacy of Baricitinib in Healing Digital Ulcers

Most of the DU were healed, and no new DU were reported, demonstrating that baricitinib is remarkably effective in improving vascular disease. It has been suggested that the use of vasodilators, particularly calcium channel blockers, may reduce the risk of the first DU in SSc patients (42). However, improvements are not always statistically significant for vasodilator therapy, such as nifedipine, sildenafil, prostaglandin E1, and iloprost, in patients with DU (43–46). In our study, two out of four patients with DU at baseline were receiving a calcium channel blocker, nifedipine, mainly for a preventive role in combination with baricitinib. Our observations suggest promising results of the efficacy of baricitinib in healing DU in dcSSc patients.

### Relevance of Lung Fibrosis

Baricitinib had a non-significant but positive clinical effect on lung outcomes in our study. Although we were could not perform lung function testing due to heightened risks associated with COVID-19 at the time of the study, our HRCT findings, together with SGRQ scores from baseline to week 24, suggested that baricitinib had a favorable clinical effect that helped slow down the progression of SSc-associated lung fibrosis and improved the respiratory symptom in SSc patients with ILD. Therefore, it warrants further evaluation with a larger dose of baricitinib.

### Interest in Baricitinib and Low-Dose Prednisone Combination for the Treatment of Skin Fibrosis

Background therapy was allowed in our study. There were three patients treated with baricitinib in combination with tapered low-dose prednisone. Notably, improvements in the mRSS in those three patients were more significant than in other patients without low-dose prednisone, suggesting a possible better benefit of baricitinib in combination with low-dose prednisone than either baricitinib or prednisone alone in skin fibrosis. Therefore, skin improvement in patients with dcSSc in our study after adding baricitinib may be due to the efficacy of baricitinib, but it may also be the effect of its combination with low-dose prednisone. However, more large clinical trials are needed to confirm our findings.

### Safety Results

Safety results were generally similar to those previously reported for patients who received tofacitinib or baricitinib in other diseases (47, 48). Herpes zoster occurred in only one patient. There were no malignant tumors, major adverse cardiovascular events, or deaths in this study.

### Limitations

This study had certain limitations. It was an open-label study with a limited number of dcSSc patients and no control group. The time frame for the interpretation of treatment effectiveness was limited to 24 weeks. The lack of lung functions and HRCT with a validated, computer-aided scoring method endpoint limited the ability to draw conclusions regarding the full effectiveness of the JAK inhibitor in ILD in this patient population. We strongly recommend that the JAK inhibitor should be tested for efficacy and safety in randomized placebo-controlled trials.

## Conclusion

Our study indicates that the JAK1 and JAK2 inhibitor has stronger antifibrotic effects than the JAK3 inhibitor, and JAK2 inhibitor use also results in the loss of capillaries in BLM-induced scleroderma mice. Furthermore, this is possibly one of the first clinical trials to specifically focus on skin fibrosis and microvascular manifestations using JAK1/2 inhibitor baricitinib in SSc. The results are promising for dcSSc patients with skin fibrosis and DU, which may be explored further with randomized-controlled trials for the treatment of fibrosing and vasculopathy disorders in general. Baricitinib might be an alternative treatment for patients with refractory dcSSc.

## Data Availability Statement

The original contributions presented in the study are included in the article/Supplementary Material, further inquiries can be directed to the corresponding authors.

## Ethics Statement

The studies involving human participants were reviewed and approved by Dermatology Hospital, Southern Medical University. The patients/participants provided their written informed consent to participate in this study. The animal study was reviewed and approved by Southern Medical University. Written informed consent was obtained from the individual(s) for the publication of any potentially identifiable images or data included in this article.

## Author Contributions

All authors designed the protocol, enrolled, and examined the patients, oversaw safety monitoring, managed and analyzed the data, and wrote the manuscript. YL and SJ designed the research studies, supervised the trial and experiment process, participated in the screening of clinical cases, evaluated the clinical effect, analyzed the results, and were involved in manuscript preparation. ZH prepared the research protocol, recruited SSc patients, collected and analyzed clinical data, and wrote the manuscript. XS conducted animal experiments, analyzed experimental results, and wrote part of the manuscript. GH recruited SSc patients, analyzed clinical data, and was involved in the manuscript preparation. RX, YaC, HW, and BY assisted with recruiting patients. YeC assisted in data collection.

## Conflict of Interest

The authors declare that the research was conducted in the absence of any commercial or financial relationships that could be construed as a potential conflict of interest.

## Publisher's Note

All claims expressed in this article are solely those of the authors and do not necessarily represent those of their affiliated organizations, or those of the publisher, the editors and the reviewers. Any product that may be evaluated in this article, or claim that may be made by its manufacturer, is not guaranteed or endorsed by the publisher.

## Supplementary Material

Supplementary Figure 1 Changes of capillary loss after the treatment with JAK inhibitor in BLM-induced skin fibrosis model on day 28 Representative H&E (scale bar: 50 μm) and immunofluorescence (scale bar: 50 μm) images stained for CD31 (endothelial cells, purple) and DAPI (nuclear staining, blue) of skin sections from PBS-treated mice exposed to vehicle **(A)**, BLM-treated mice exposed to vehicle **(B)**, and BLM-treated mice receiving 30 mg/kg/day JAK2 inhibitor **(C)** on day 28. Three replicate experiments were conducted for a sum of six mice per group.

Supplementary Figure 2 HRCT in nine participants from baseline to week 24 **(A–I)**.
