# Corrigendum: JAK1/2 inhibitor baricitinib improves skin fibrosis and digital ulcers in systemic sclerosis

**DOI:** 10.3389/fmed.2022.1125836

**Published:** 2023-01-05

**Authors:** Zhanying Hou, Xuehan Su, Guangming Han, Ruzeng Xue, Yangxia Chen, Ye Chen, Huan Wang, Bin Yang, Yunsheng Liang, Suyun Ji

**Affiliations:** ^1^Department of Dermatology, Dermatology Hospital, Southern Medical University, Guangzhou, China; ^2^Department of Dermatology, Shenzhen Longhua District Central Hospital, Shenzhen, China; ^3^The First School of Clinical Medicine, Southern Medical University, Guangzhou, China; ^4^Department of Rheumatology, Dermatology Hospital, Southern Medical University, Guangzhou, China

**Keywords:** baricitinib, JAK inhibitor, systemic sclerosis, skin fibrosis, digital ulcers

In the published article, there was an error regarding the affiliations for author “Xuehan Su.” As well as having the affiliation “Department of Dermatology, Dermatology Hospital, Southern Medical University, Guangzhou, China,” they should also have “The First School of Clinical Medicine, Southern Medical University, Guangzhou, China.”

The authors apologize for this error and state that this does not change the scientific conclusions of the article in any way. The original article has been updated.

