# Supplementary figures and images for: JAK1/2 Inhibitor Baricitinib Improves Skin Fibrosis and Digital Ulcers in Systemic Sclerosis
Source: Front Med (Lausanne). 2022 Jun 6;9:859330. doi: 10.3389/fmed.2022.859330 (PMC9208297; doi:10.3389/fmed.2022.859330)

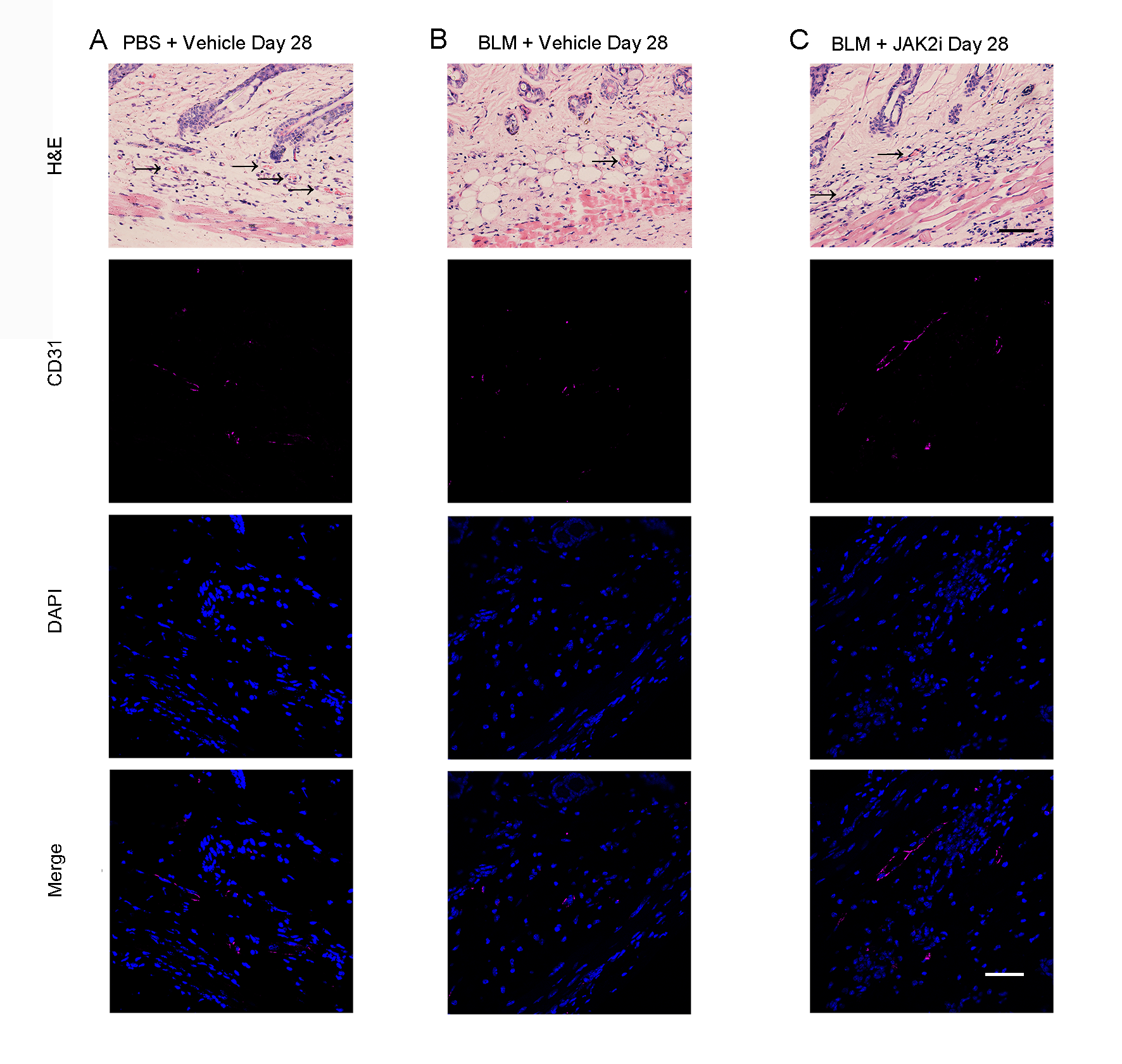

Supplement: Supplementary Figure 1 — Changes of capillary loss after the treatment with JAK inhibitor in BLM-induced skin fibrosis model on day 28 Representative H&E (scale bar: 50 μm) and immunofluorescence (scale bar: 50 μm) images stained for CD31 (endothelial cells, purple) and DAPI (nuclear staining, blue) of skin sections from PBS-treated mice exposed to vehicle (A), BLM-treated mice exposed to vehicle (B), and BLM-treated mice receiving 30 mg/kg/day JAK2 inhibitor (C) on day 28. Three replicate experiments were conducted for a sum of six mice per group. [file Image_1.TIF]
