# Supplementary material for: JAK1/2 Inhibitor Baricitinib Improves Skin Fibrosis and Digital Ulcers in Systemic Sclerosis
Source: Front Med (Lausanne). 2022 Jun 6;9:859330. doi: 10.3389/fmed.2022.859330 (PMC9208297; doi:10.3389/fmed.2022.859330)

A.

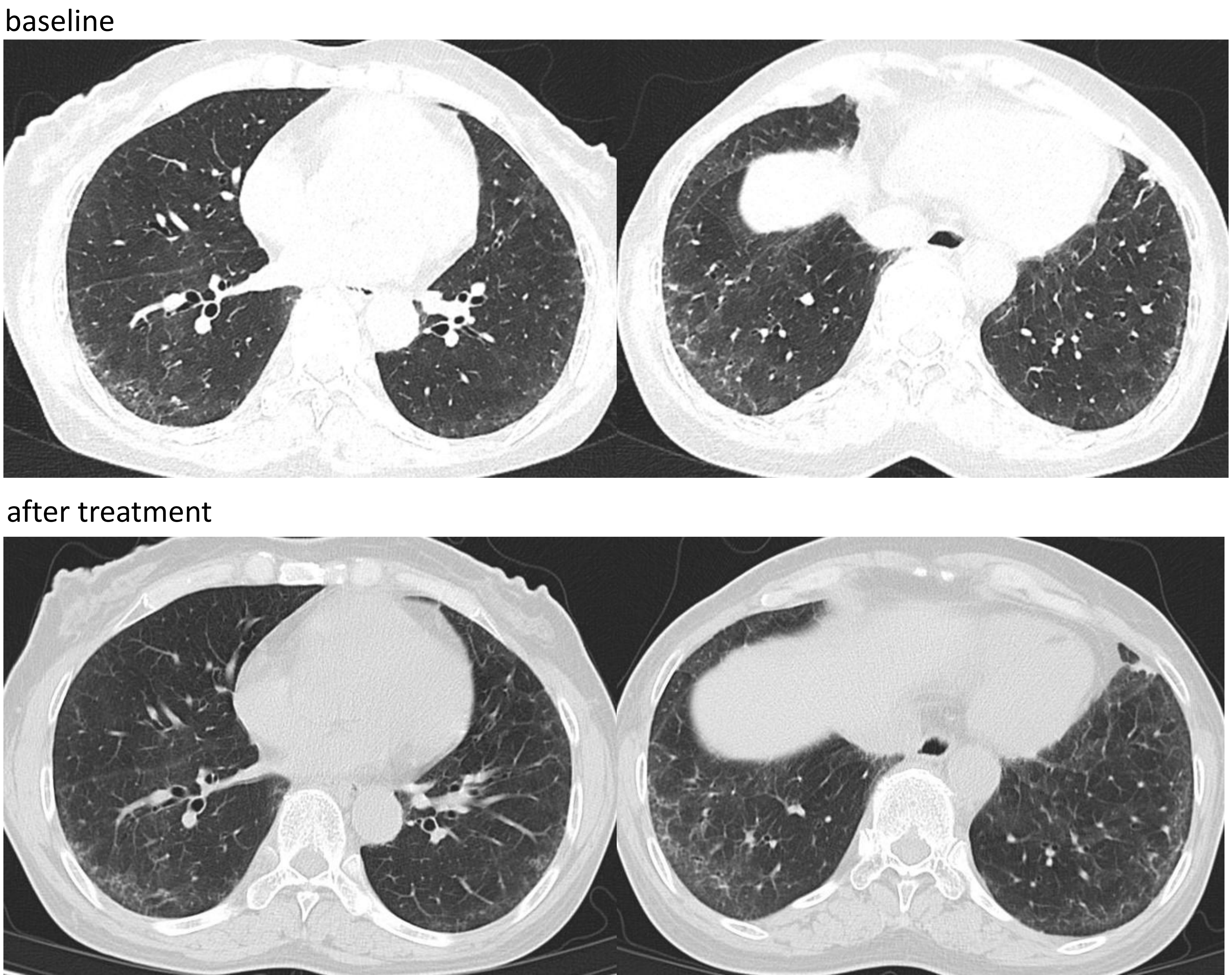

B. baseline

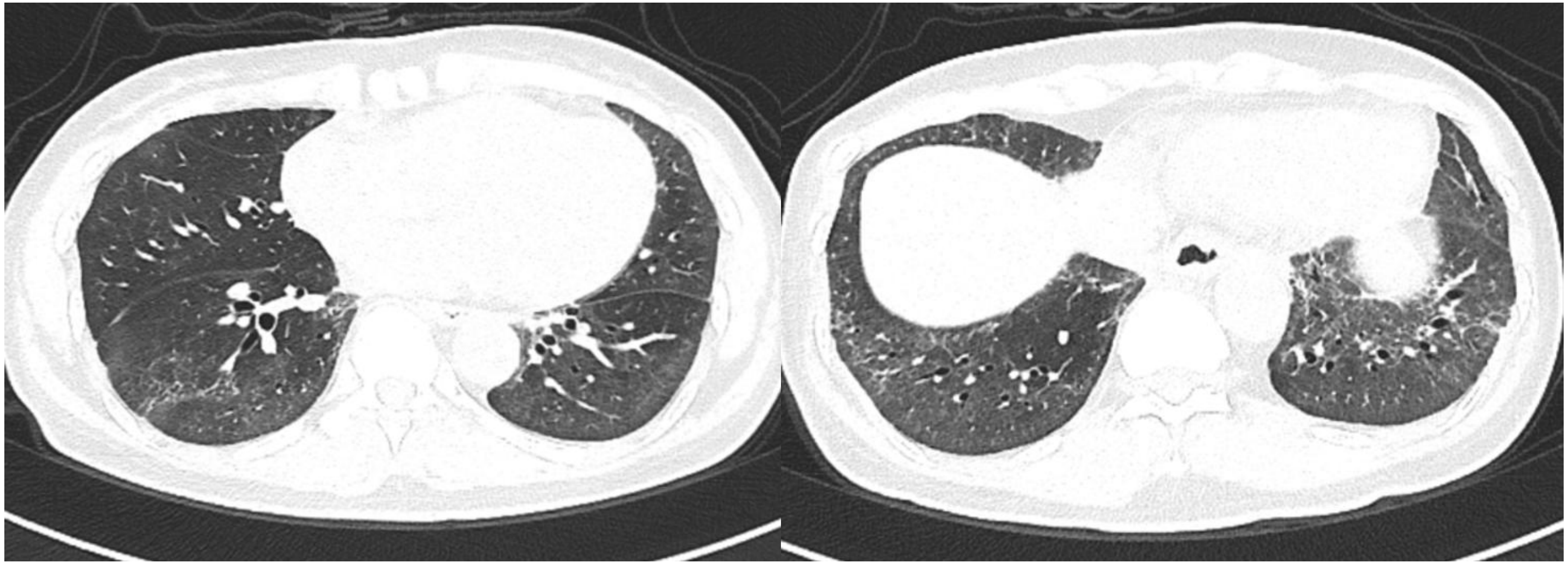

after treatment

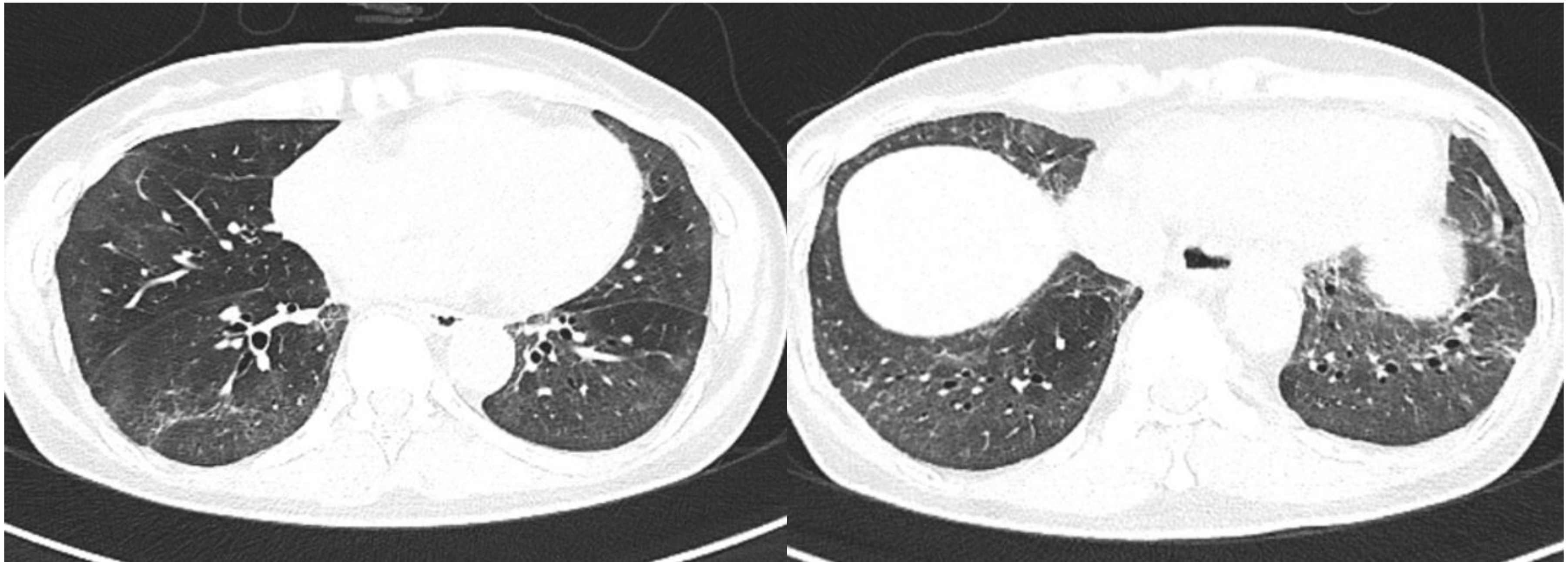

C. baseline

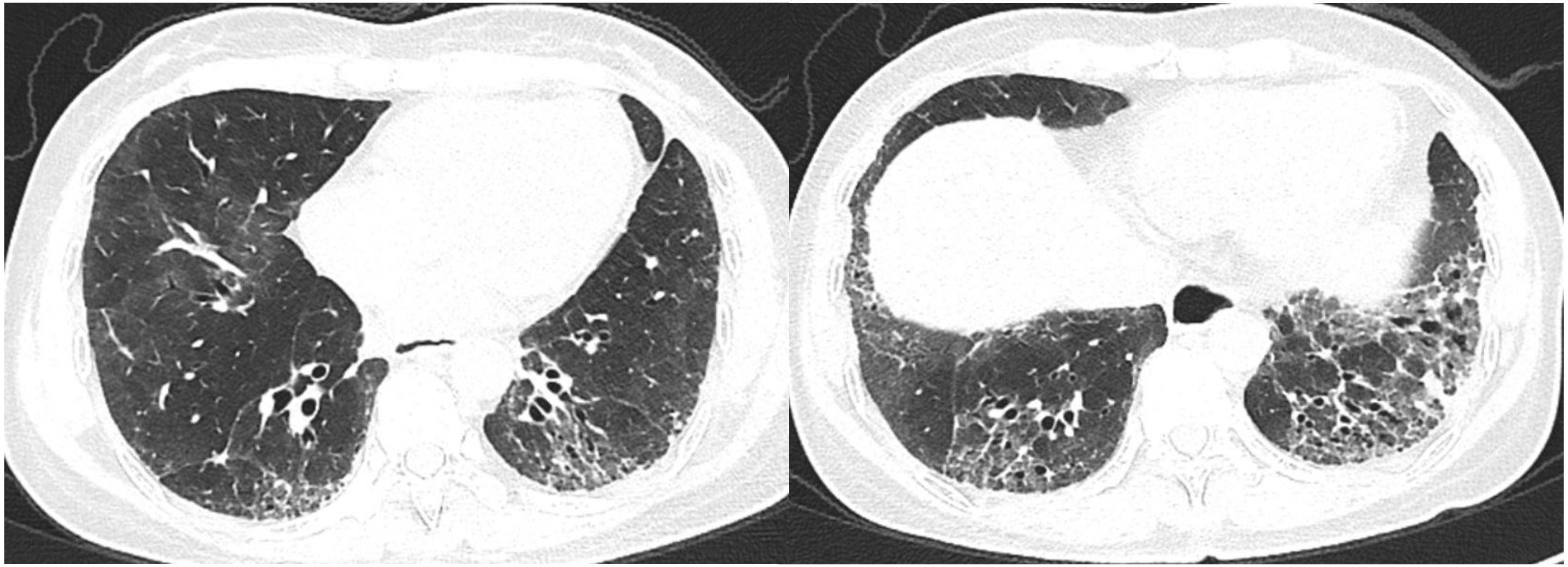

after treatment

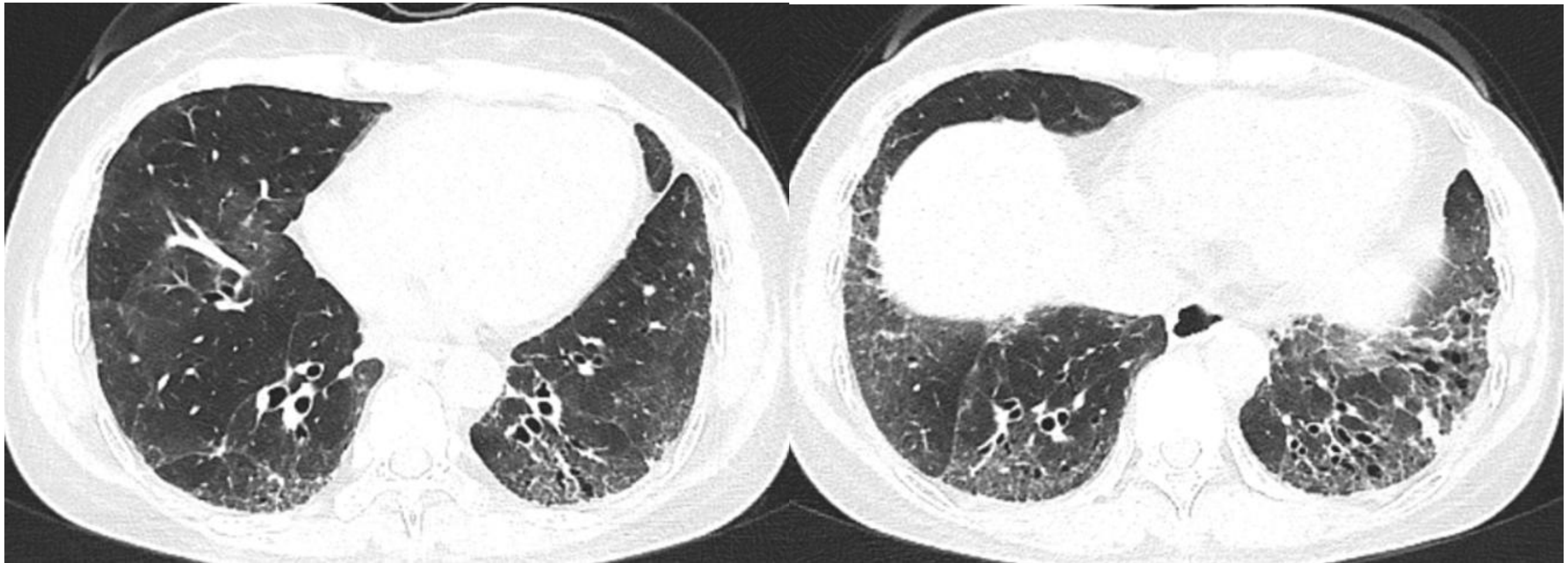

D. baseline

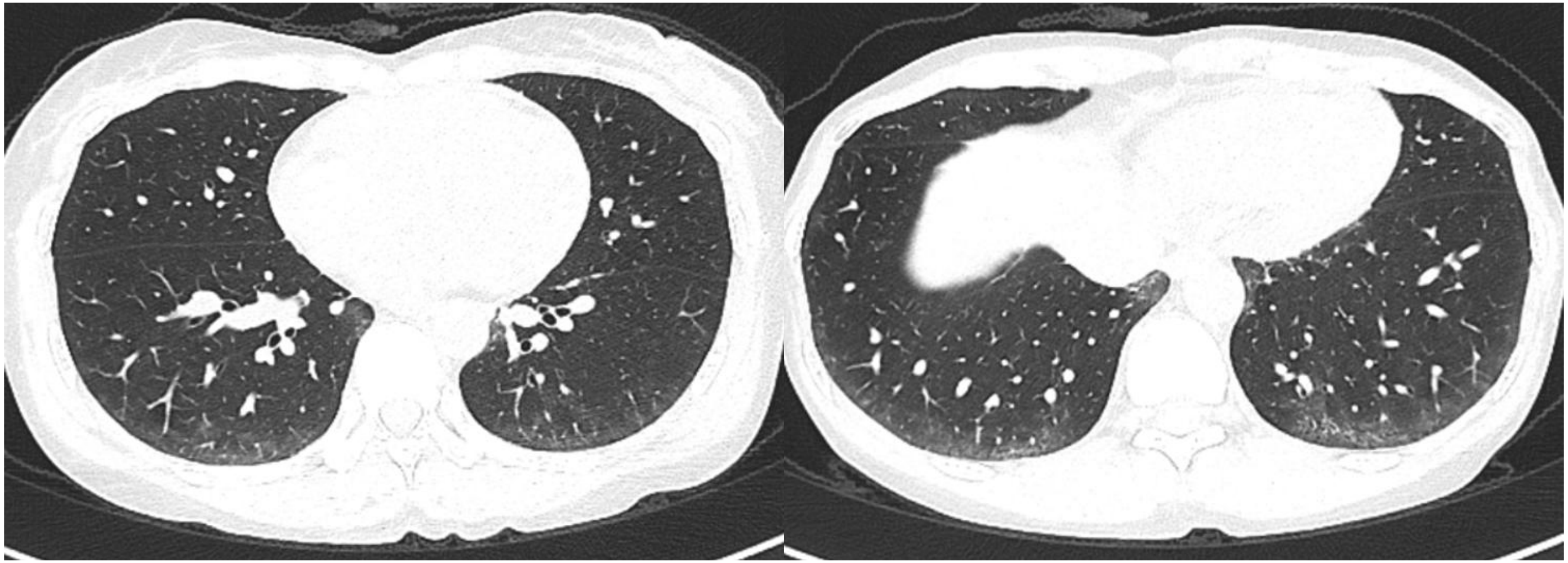

after treatment

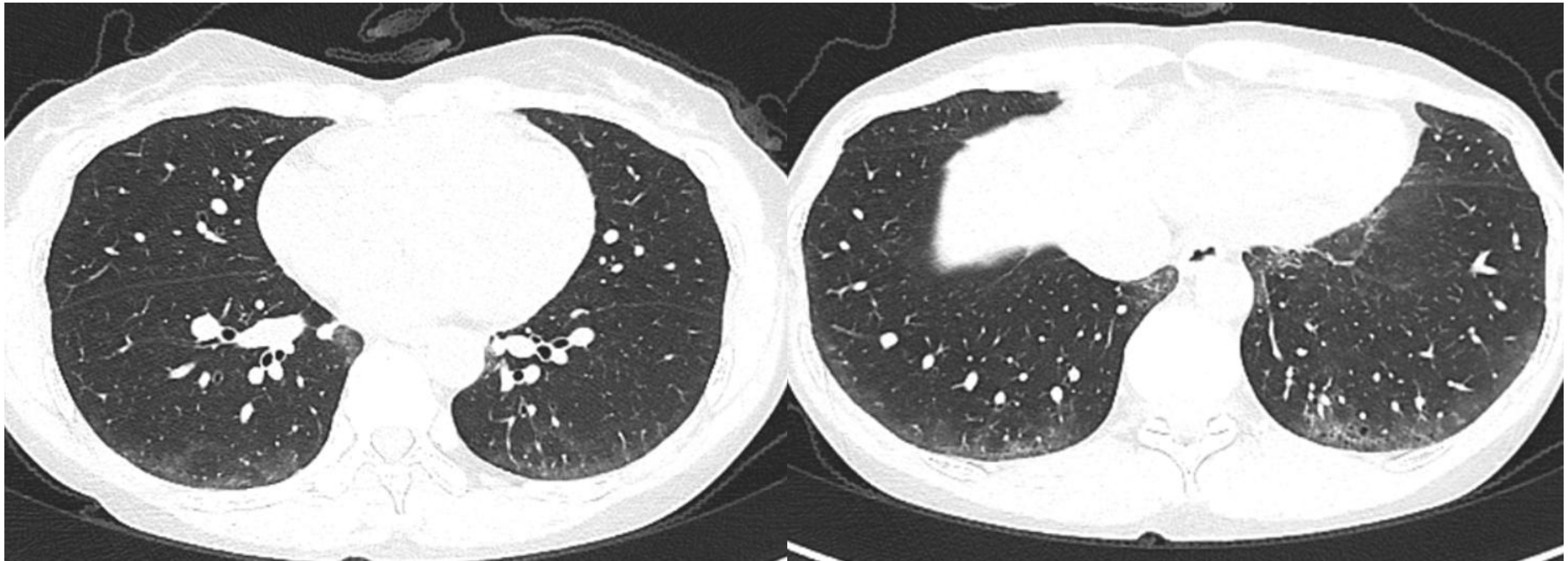

E. baseline

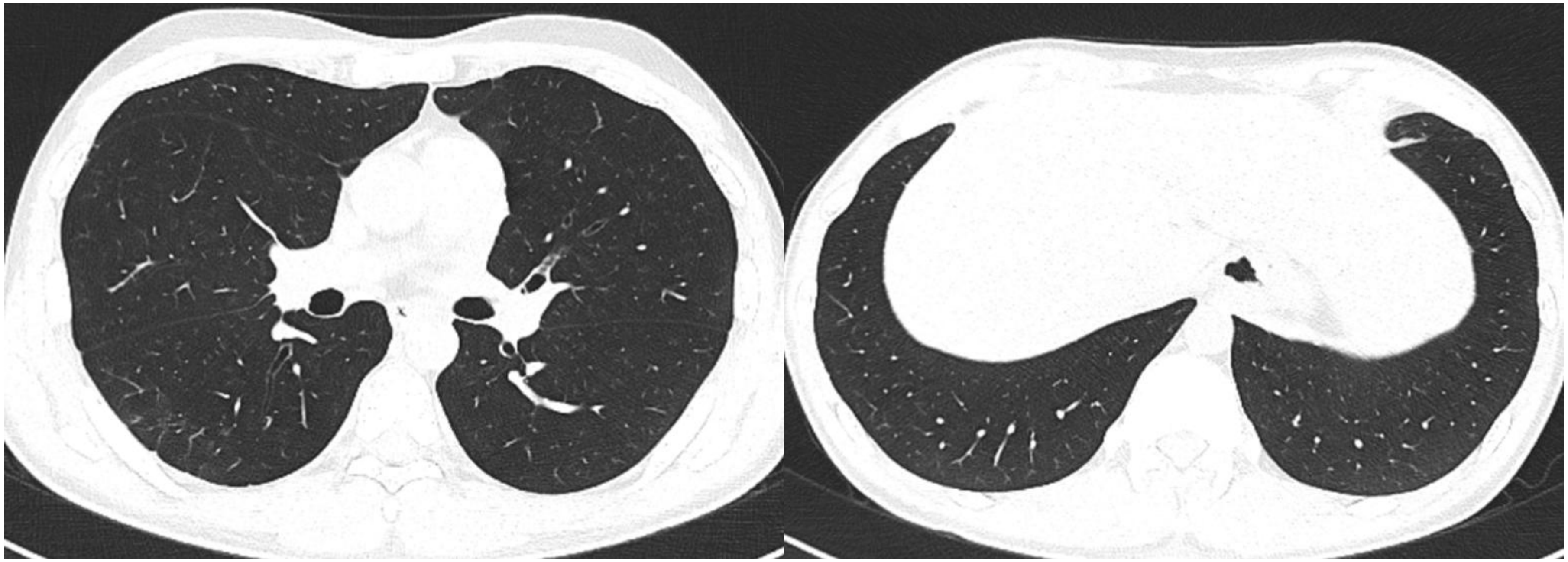

after treatment

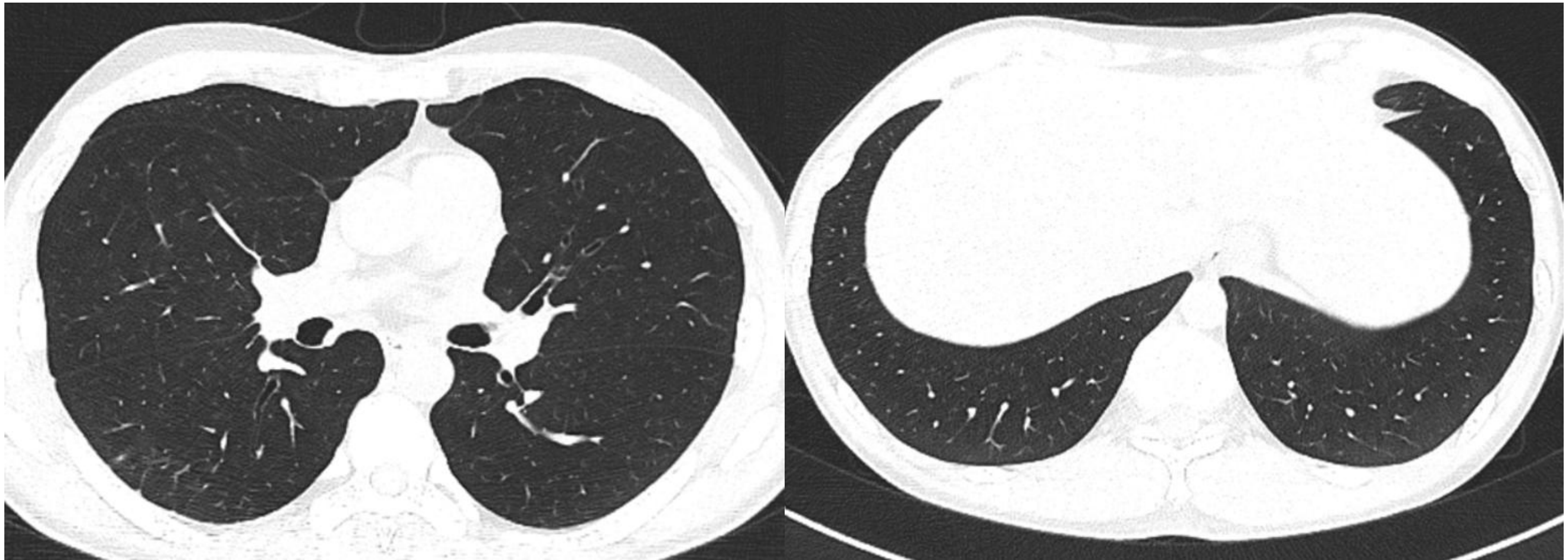

F. baseline

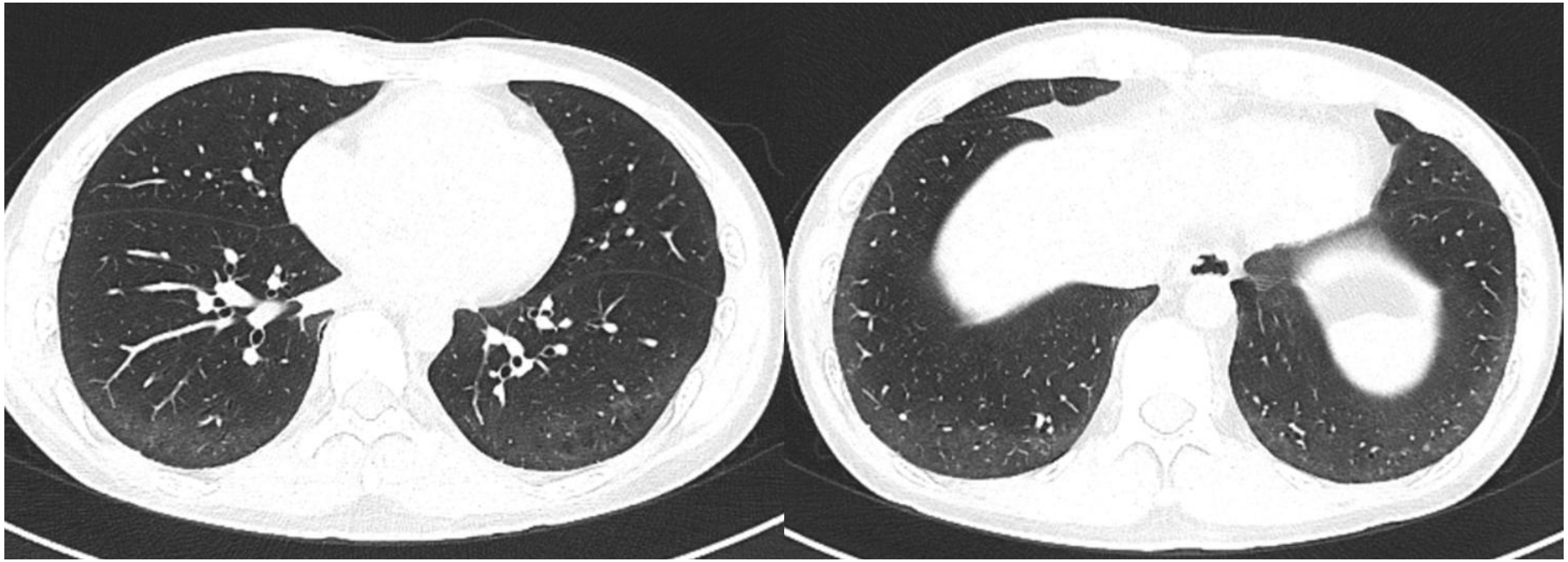

after treatment

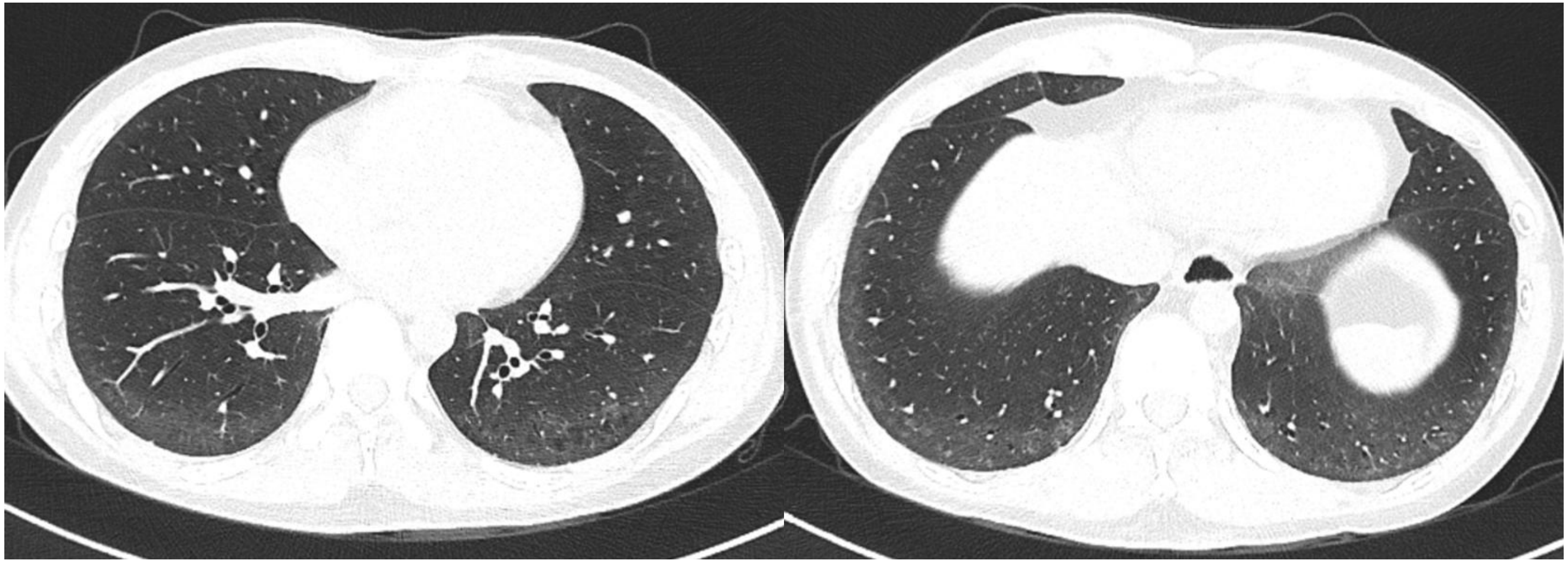

G. baseline

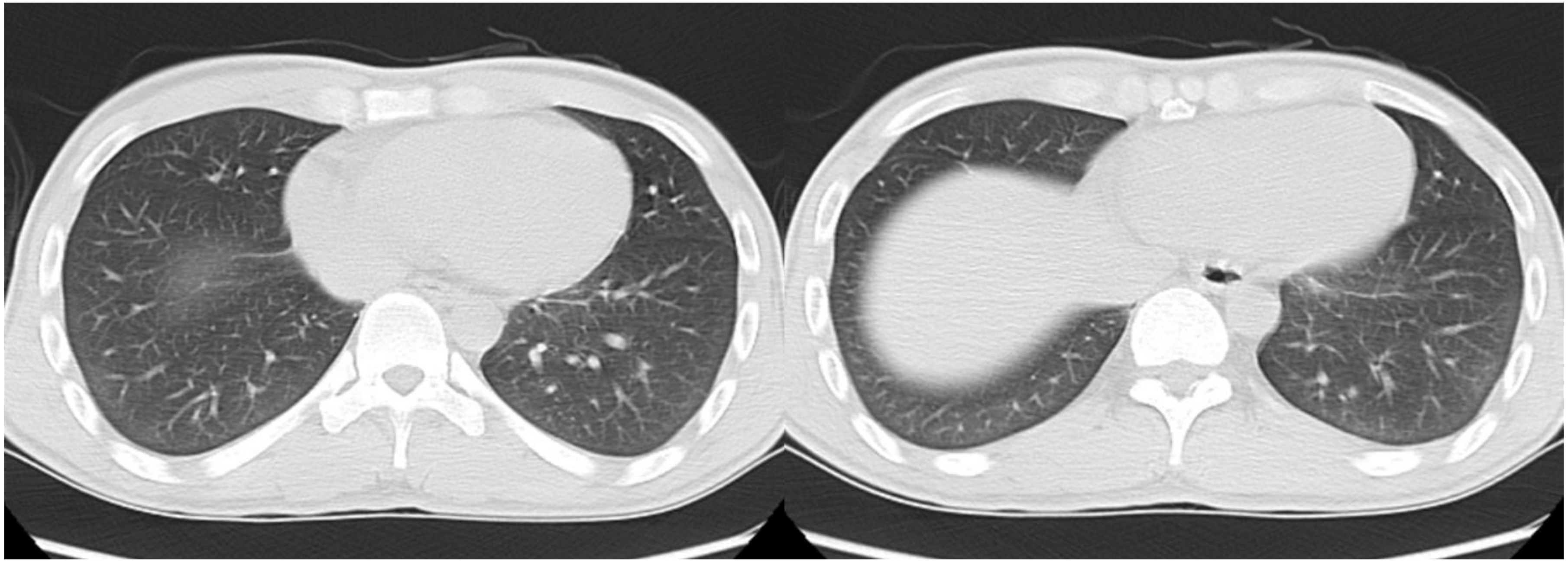

after treatment

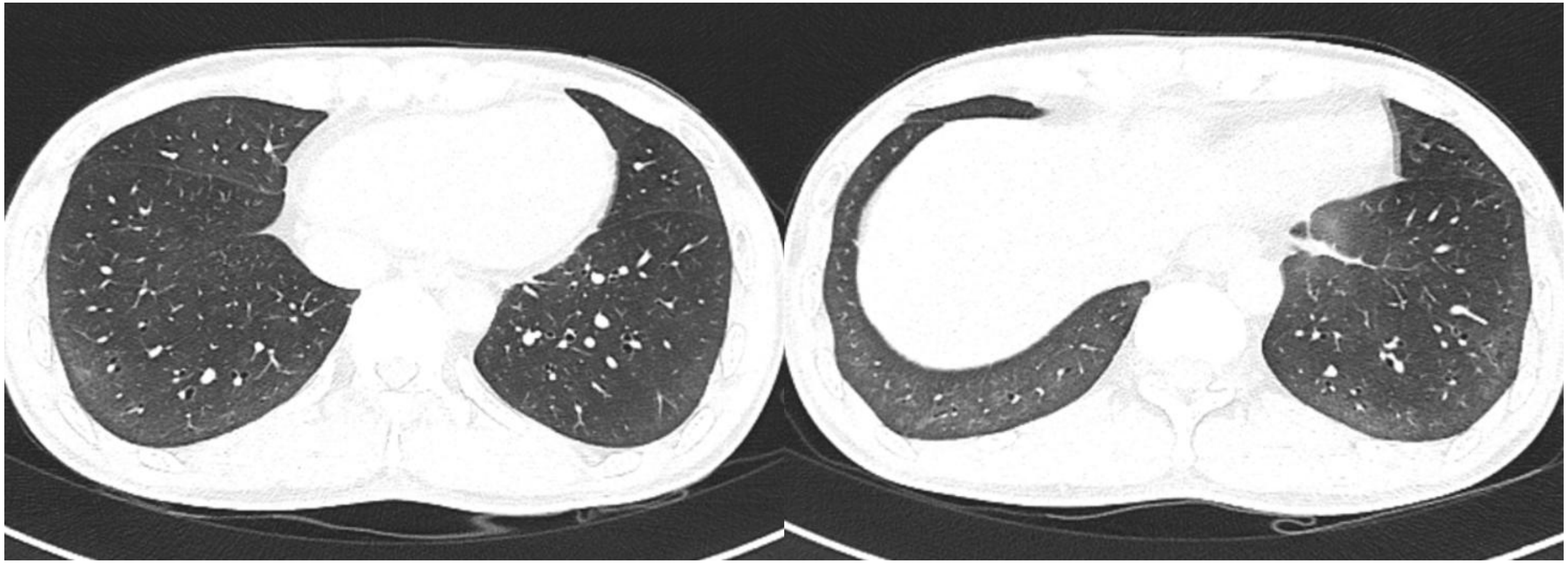

H. baseline

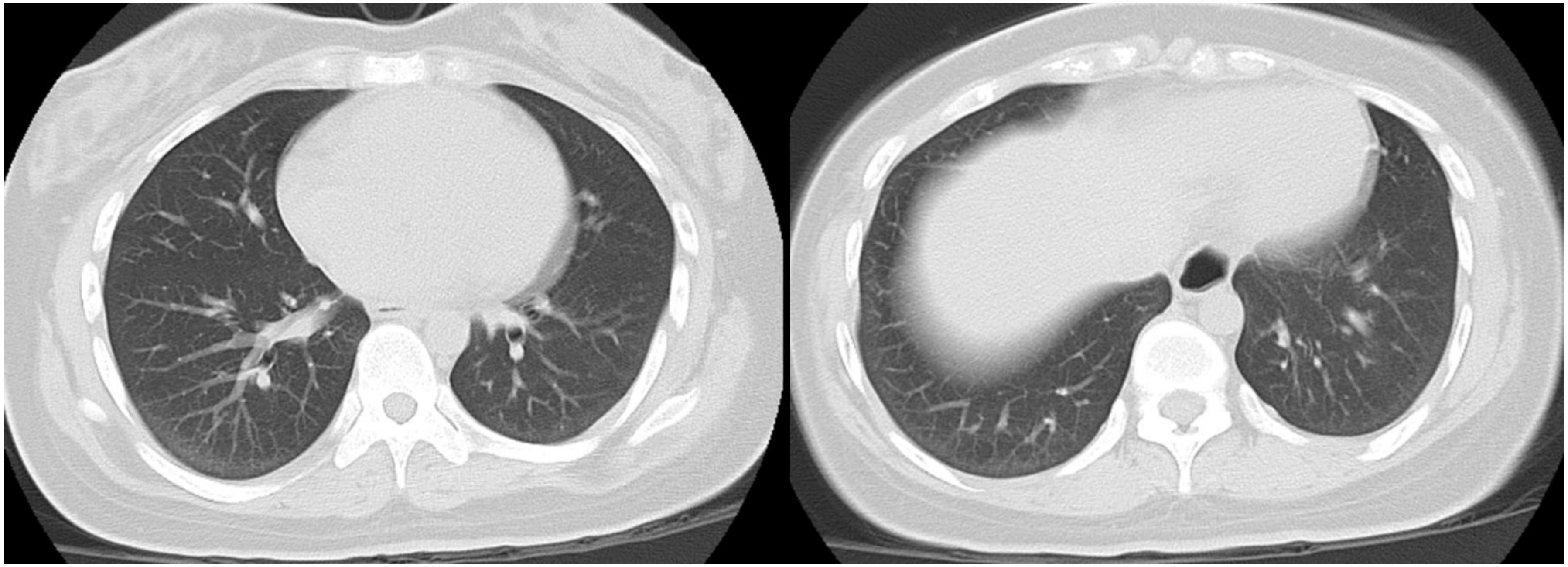

after treatment

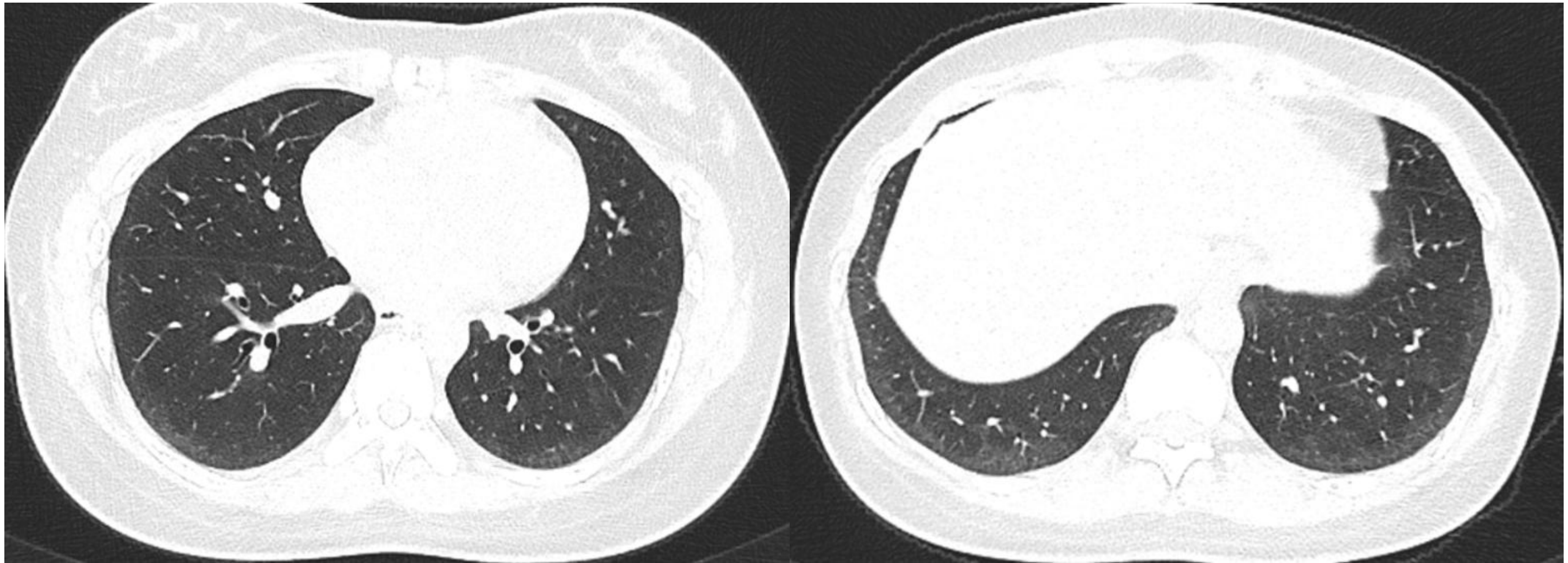

I. baseline

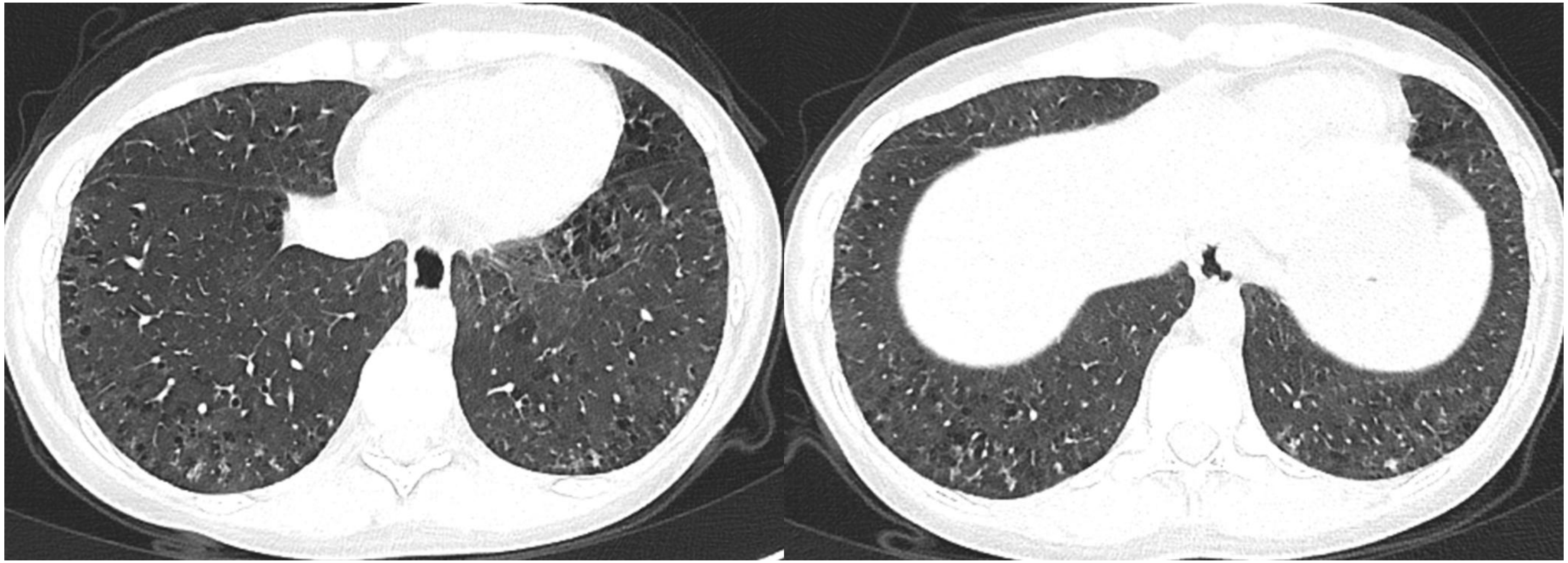

after treatment

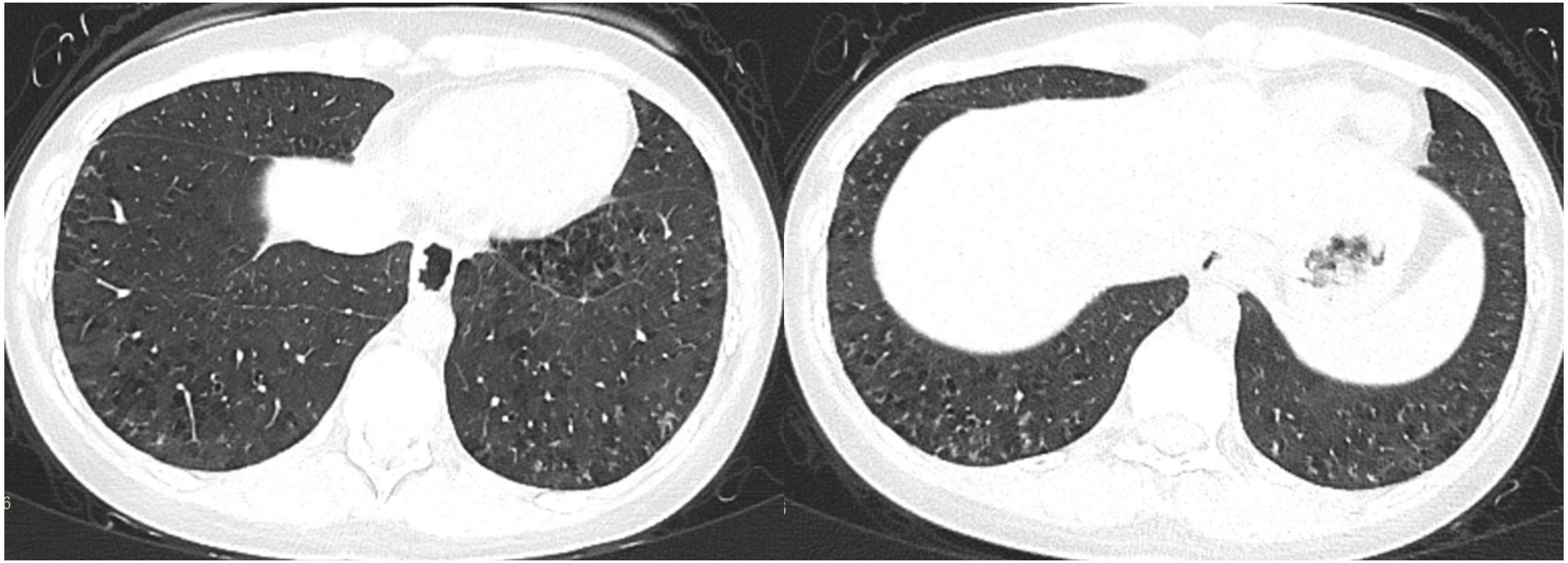

Supplement: Supplementary Figure 2 — HRCT in nine participants from baseline to week 24 (A–I). [file Data_Sheet_1.PDF]
